# Experience in Managing a Complex Case of Infected Achilles Tendon Rupture with Segmental Loss: A Case Report

**DOI:** 10.5704/MOJ.2507.017

**Published:** 2025-07

**Authors:** MS Ahmad-Ismani, JS Chong, MA Hj-Salleh, MAS Ayeop, A Che-Ahmad

**Affiliations:** Department of Orthopaedics, Sultan Ahmad Shah Medical Centre, Kuantan, Malaysia

**Keywords:** achilles tendon rupture, achilles tendon segmental loss, Baker’s U-lengthening

## Abstract

The Achilles tendon rupture often present as a significant clinical challenge. We present a complex case of chronic Achilles tendon rupture complicated by infection and segmental loss after failed primary repair. The patient underwent meticulous debridement, reconstruction using Baker's U-lengthening technique with augmentation using plantaris tendon. Tension-relieving sutures and a biosynthetic graft [Artelon® Flexband] were used for enhanced support. Post-operatively, a structured rehabilitation program was implemented, leading to successful healing with full weight-bearing capability at 12 weeks, with improved ankle function and no evidence of contracture. The utilisation of Baker's U-lengthening, along with adjunctive measures, proved effective in managing this challenging case. This report highlights the importance of a multidisciplinary approach, incorporating orthopaedic, infectious disease, and rehabilitation specialists in the comprehensive management of complex Achilles tendon injuries.

## Introduction

Achilles tendon is an important tendon at the back of the heel which is only covered by a thin layer of soft tissue distally. It is the largest but also the most common tendon to rupture^1,2^. The management of Achilles tendon injury can be challenging in presence of infection, tendon segmental loss and defect in skin coverage. Respect to soft tissue is detrimental to avoid wound breakdown and infection. There are different methods of tendon reconstruction and augmentation described to manage segmental loss of tendon substance.

We present a difficult case of chronic Achilles tendon rupture with failed primary repair complicated by infection and segmental loss of tendon substance.

## Case Report

We present a 58-year-old man working as a helicopter pilot with underlying hypertension, dyslipidaemia, and obesity. He heard a pop sound behind his right ankle when trying to manoeuvre his motorcycle onto a double stand using his forefoot with ankle plantar flexed. Post trauma, he sustained posterior ankle pain and progressive weakness on moving his ankle. He sought medical attention at another hospital six months post injury and was diagnosed as right distal Achilles tendon rupture. Achilles tendon repair was done using anchor suture but unfortunately, it was subsequently complicated by surgical site infection. Wound debridement was done, antibiotic started, and patient underwent regular dressing for two months. He was then referred to our centre for further management at 11 months post trauma.

Upon local examination, noted a 5cm x 2cm wound at the posterior aspect of right ankle. A friable proximal stump of Achilles tendon seen with loss of substance distally. Otherwise, the wound base is healthy and there were no active signs of infection. Plantar flexion power is grade three. Passive range of motion the ankle is normal with 10° dorsiflexion and 45° plantarflexion. Simmonds-Thompson test is positive. Bedside ultrasound revealed loss of Achilles tendon substance with 2cm of distal stump remaining. Plain radiographs showed no abnormalities.

**Fig. 1:**
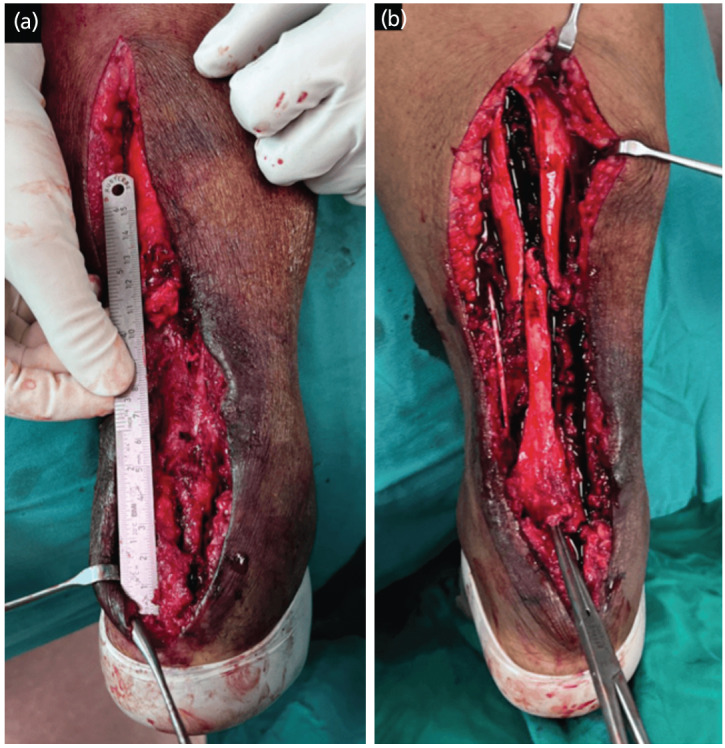
(a) Wound after debridement and freshening of Achilles tendon ends. Proximal stump is 11cm away from its distal insertion at the posterior calcaneum. 2cm distal stump over medial side was still intact. (b) Baker’s U-lengthening done, and tendon slide down distally. Subsequently. the proximal tenotomy site was repaired using Ethibond suture, and the distal end of mobilised tendon was fixed onto the decorticated calcaneum using fibre-wire and anchor suture.

**Fig. 2:**
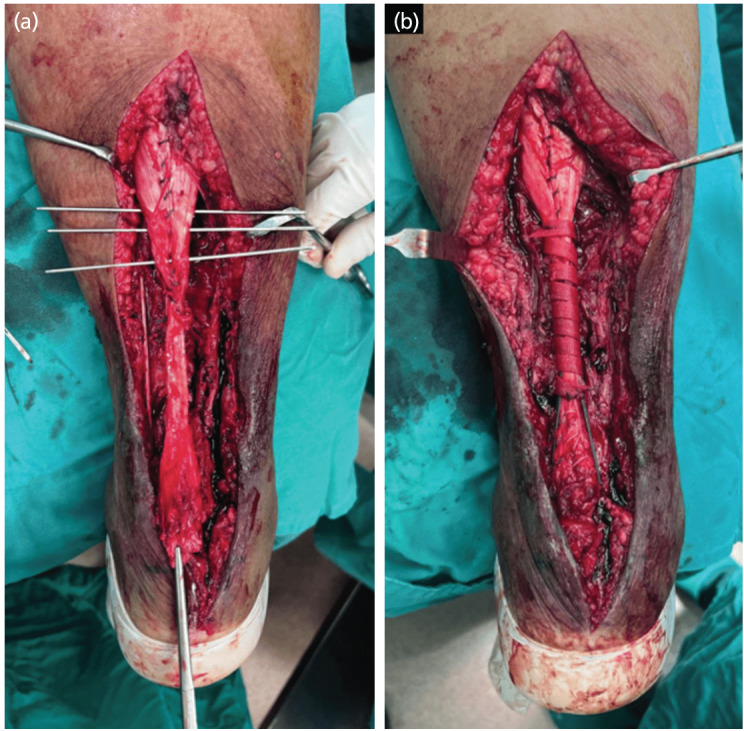
(a) Three tension-relieving polyester sutures [Ethicon ETHIBOND EXCEL] inserted to the proximal tendon using blunt needle and are anchored distally to either side of calcaneum using suture anchors. Forceps at the bottom was holding the intact distal stump which was sutured onto the mobilised distal segment of Baker’s U lengthening. (b) A biosynthetic graft [Artelon® Flexband] was whorled and sutured to the repaired tenotomy site containing the interweaved plantaris tendon.

The patient was positioned prone after giving combined spinal epidural anaesthesia, painting and draping were done and tourniquet was inflated. Intra-operatively, wound was curetted and irrigated, inverted wound edges debrided. A lazy-S incision was extended proximally and distally. Suture and loosed anchor screw from the previous surgery were removed. The unhealthy and friable ends of Achilles tendon was debrided. After freshening of tendon ends, the proximal tendon is 11cm away from the insertion at calcaneus, 2cm medial half of distal stump was intact (Fig. 1a). The proximal part of Achilles tendon was exposed and mobilised up to the sufficient length. Baker U-lengthening was performed using size 15 blade. The distal segment of aponeurotomy was moved distally and fixed onto the calcaneum using a 4-limbs suture anchor with fibre wires, and the remaining intact distal Achilles stump was sutured onto the anchored distal segment to provide additional support (Fig. 1b). Proximally, the tendon edges were sutured using fibre-wire. Next, tension relieving sutures using polyester sutures [Ethicon ETHIBOND EXCEL] were passed through the proximal tendon and the limbs at each side anchored onto the calcaneus using suture anchor (Fig. 2a). Plantaris tendon at the medial side was skeletonised and transected proximally, then weaved over the junction of repair to augment it. A biosynthetic graft [Artelon® Flexband] is whorled and sutured over the proximal repair site before wound closure (Fig. 2b). Finally, the ankle was immobilised at gravity equinus using a dorsal blocking slab.

**Fig. 3:**
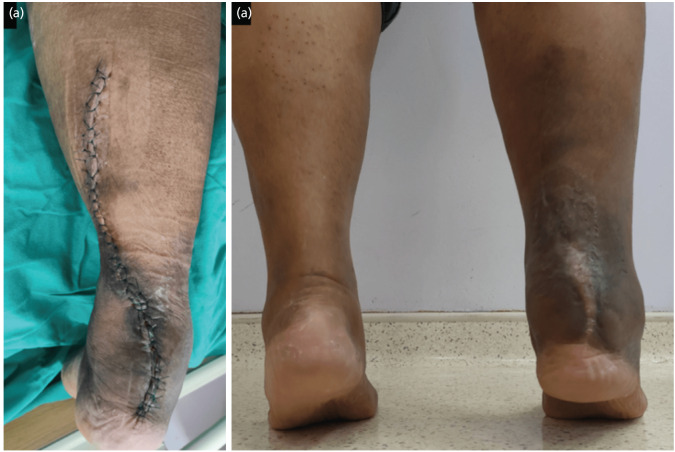
(a) Wound inspection on day 2 post-operation. (b) At 12 weeks post-operation, patient was able to full weight bear with good plantar flexion power.

Post-operatively, patient was kept in ward for daily dressing and intravenous antibiotic until the wound is dry (Fig. 3a). During his follow-up visit at three weeks post-operatively, the wound was clean and skin sutures removed. Dorsal slab was changed to a hinged ankle brace and locked at neutral plantigrade position until six weeks post-operation, which was then unlocked to allow range of motion exercises and partial weight bearing ambulation. At 12 weeks post-operation, patient started to full weight bear. The wound was well healed with no contracture. The patient was able to actively dorsiflex 10° and plantarflex the ankle to 45° with clinically no reduced power compared to the opposite side (Fig. 3b). The ankle brace was off, and he was advised for footwear with a heel raise insert until six months post-operation.

## Discussion

Achilles tendon rupture can be caused by direct injuries such as cuts to the back of the heel or impact on a tensed tendon, or indirect injuries resulting from sudden stretching during dorsiflexion or forceful dorsiflexion in a plantar-flexed ankle. These injuries commonly occur in people in their 30s to 50s, especially among those who engage in occasional physical activity, known as “weekend warriors”^1,2^. Other risk factors include inadequate conditioning before exercise, overexertion, prior Achilles tendinopathy, extended use of corticosteroids, fluoroquinolone antibiotics, oral bisphosphonates, diabetes, hyperparathyroidism, and genetic predisposition^2^.

Achilles tendon injury is primarily diagnosed based on clinical evaluation, with imaging providing confirmation and additional details. Magnetic resonance imaging (MRI) or ultrasonography can be used for confirmation, offering 100% sensitivity^1,3^. However, MRI may not accurately distinguish between partial and complete ruptures, while ultrasonography is more effective at locating a tear, assessing the gap between torn tendon ends, and differentiating between partial and complete ruptures^1^. Additionally, ultrasound allows for dynamic observation of tendon gaps, which strongly correlate with observations made during surgical repair. Some experts suggest that a gap greater than 5mm between tendon ends in a full equinus position, as seen on ultrasound, indicates the need for surgical intervention^3^. Lateral ankle plain radiographs help identify tendon swelling and increased soft tissue density in the Kager's fat pad. They are particularly useful in detecting calcific lesions, Haglund's prominence, and calcaneus avulsion fractures, all of which may indicate pre-existing degeneration or chronic tendinosis. A lateral view radiograph showing an anteroposterior Achilles tendon width of 8mm or more may suggest a pathological tendon^4^.

Infection is a serious complication that can occur in open injuries or as a complication following primary repair, often presenting with loss of the overlying skin and tendon tissue. Proper management involves thoroughly debriding the wound to healthy bleeding edges and removing any infected or degenerated tendon tissue. The wound should be managed by moist dressing until a clean, healthy bed is established before proceeding with definitive procedure. Options for soft tissue coverage include reverse sural artery flap, perforator-based propeller flaps, medial plantar flap with plantar aponeurosis, reverse peroneus brevis flaps, and free flaps such as anterolateral thigh or latissimus dorsi flaps, which require microvascular expertise^4^.

Conservative management is a feasible approach for treating acute Achilles tendon ruptures, but the re-rupture rate is 2 to 3 times higher with conservative treatment (8-13%) compared to surgical repair (4%)^3,5^. Additionally, conservative treatment may lead to potential loss of strength and an increased risk of tendon adhesion^3^. Surgical intervention more effectively reduces the risk of re-rupture, however, is associated with few surgical complications^5^. Major complications include wound infection, fistula formation, skin necrosis, suture granuloma, and sural nerve damage. Skin necrosis in the back of the ankle can lead to significant morbidity, necessitating complex plastic surgery procedures for adequate tendon coverage^5^.

For chronic Achilles tendon injuries, surgery is the primary treatment option. Generally, primary end-to-end repair can be attempted for full-thickness defects of less than 2cm. A gap of 2cm to 5cm requires tendon lengthening with or without augmentation. Gaps greater than 5cm are more complex and may necessitate tendon lengthening combined with a tendon transfer and potentially an allograft^4^. In this particular case, the chosen tendon lengthening method was Baker’s inverted U or 'tongue-in-groove' technique, which allowed us to recover 11cm of tendon gap. This technique was chosen due to our familiarity with this procedure and its ability to manage large tendon gap. Other tendon lengthening techniques include slide lengthening via triple hemisection, V-Y plasty, Z-plasty, Vulpius gastrocnemius-soleus recession, gastrocnemius turndown flap, and Lindholm technique. Regrettably, despite an extensive literature search, we found that many journals focus on comparing these methods as tendon lengthening techniques rather than their application in the repair of segmental tendon loss. Apart from a few case reports demonstrating success with individual techniques, there is a lack of comprehensive comparative studies on the various salvage methods used to repair Achilles tendon segmental loss. Hence this recommends the need for larger, comparative studies to guide best practices in the future.

In the event in which lengthening cannot be done, tendon transfer could be performed to reconstruct the Achilles tendon. Flexor hallucis longus (FHL) tendon transfer is the preferred approach because the muscle is synergistic and the strongest of the available options. The FHL muscle and tendon have demonstrated up to 52% hypertrophy after transfer, showing a strong capacity for adaptation. Over time, the concentric and eccentric contraction strength of the FHL transfer can match that of the normal side^2,4^. An alternative option is the peroneus brevis tendon transfer, where the tendon is harvested through a distal incision and retrieved through the surgical wound behind the fibula. The tendon is inserted into the calcaneus in a dorsoplantar direction using an interference screw and can also be connected to the distal stump of the Achilles tendon. Free tendon grafts such as the semitendinosus tendon, gracilis tendon, and fascia lata may be used in cases where reconstruction with tendon flaps is not feasible^4^.

Reconstruction or primary repair of the Achilles tendon can be augmented using a portion of its own tendon or other tendons such as the plantaris, flexor hallucis longus, and peroneus brevis. In plantaris tendon augmentation, the tendon is stripped and transected proximally, then either woven around the Achilles tendon or spread into a membrane that is sutured around the repair^2,3^. Additionally, our repair was enhanced using Artelon® Tissue Reinforcement FLEXBAND, a synthetic, degradable polyurethane urea material designed to provide extra mechanical strength to the tendon repair while serving as a degradable scaffold that integrates with the patient's tissue. It stretches and rebounds similarly to natural ligaments and tendons, facilitating motion while maintaining stability. This material quickly integrates into healing tissue in the short term, then slowly dissolve and fully integrate over few years. This process of integration increases strength, elasticity, and the biological restoration of tendon tissue. Artelon FLEXBAND is approved by the United States Food Drug Administration (FDA) for reinforcement of tendon repair including rotator cuff, patellar, Achilles, biceps, or quadriceps tendons.

Post-operative rehabilitation is a crucial part of the recovery process after reconstructing the Achilles tendon. Initially, the ankle should be kept in equinus for around two weeks, followed by a neutral plantigrade position^4^. At six weeks, the patient can begin range of motion exercises and is allowed protected partial weight-bearing using a heel raise or hinged ankle-foot orthosis^3^. Historically, Achilles tendon repair was protected for an extended period to ensure proper healing. However, early range of motion exercises have been shown to improve isokinetic calf strength and shorten the rehabilitation time compared to longer immobilisation^3^. Given that tendon healing is responsive to mechanical stimuli, range of motion exercises and protected weight-bearing should not be postponed beyond six weeks.

In conclusion, infected Achilles tendon rupture occurs due to an open injury or following a failed primary repair. It is associated with soft tissue loss involving the tendon and skin. Prior to tendon reconstruction, the infection should be treated with surgical debridement, antibiotics, and regular dressing. Baker’s U-lengthening technique is a reliable to manage tendon segmental loss with large tendon gap. The repair could be augmented by using tendon graft such as plantaris. Synthetic polyurethane urea sheet or band could be used to provides additional support and promotes tendon healing. Post-operative rehabilitation with range of motion exercise and protected weight bearing by six weeks post-operation is imperative to ensure good outcome. Multidisciplinary approach should be utilised, involving orthopaedic surgeon, a rehabilitation specialist, sports physician, and physiotherapist.
